# Supplementary figures and images for: ALDH3A2, ODF2, QSOX2, and MicroRNA-503-5p Expression to Forecast Recurrence in TMPRSS2-ERG-Positive Prostate Cancer
Source: Int J Mol Sci. 2022 Oct 2;23(19):11695. doi: 10.3390/ijms231911695 (PMC9569942; doi:10.3390/ijms231911695)

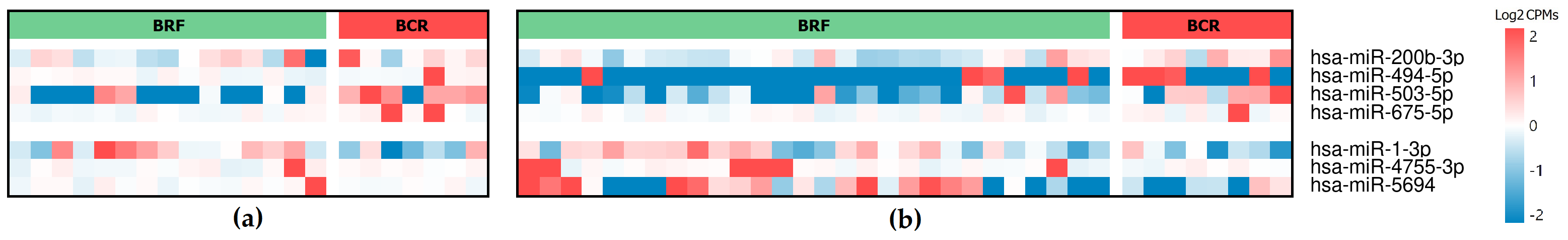

Supplement: Supplementary file 1 [file ijms-23-11695-s001.zip › Supplementary_Figure_S1_top7.png]
